# Exploring similarities and differences between *Toxoplasma gondii* and *Neospora caninum* infections in dogs

**DOI:** 10.1007/s11259-024-10549-z

**Published:** 2024-09-25

**Authors:** Giulia Morganti, Giulia Rigamonti, Leonardo Brustenga, Valentina Calgaro, Giovanni Angeli, Iolanda Moretta, Manuela Diaferia, Fabrizia Veronesi

**Affiliations:** Department of Veterinary Medicine, University of Perugia, Perugia, Italy

**Keywords:** *Toxoplasma gondii*, *Neospora caninum*, Dog, Similarities, Differences

## Abstract

*Toxoplasma gondii* and *Neospora caninum* infections in dogs are predominantly manifest asymptomatic. However, these infections can also present highly varied and potentially severe clinical signs. This is due to the parasites’ ability to replicate in a number of cell types within the host organism, with *N. caninum* exhibiting a particular tropism for the central and peripheral nervous systems, and *T. gondii* targeting the central nervous system and musculature. In clinical practice, toxoplasmosis and neosporosis are often considered to be closely related diseases, despite their distinct epidemiological, clinical, diagnostic, and therapeutic characteristics. The present review analyses the similarities and differences between these two protozoan infections, since an accurate and timely aetiological diagnosis is essential for establishing effective therapeutic protocols and control strategies.

## Introduction

*Toxoplasma gondii* and *Neospora caninum* are two obligate intracellular parasites belonging to the phylum Apicomplexa and the order Eucoccidiorida. Unlike the correctly defined coccidia (e.g. *Cystoisospora canis*,* Cystoisospora ohioensis* complex), they are not included in the aetiological agents capable of causing enteritis in dogs. In fact, their clinical impact is not related to the intestinal development phase (present only in *N. caninum*), but rather to the extraintestinal phase that develops in multiple organs, with elective tropism for the nervous system (Black and Boothroyd 2000; Dubey et al. 2007).

Both parasites play an important role in veterinary medicine. They are responsible for serious clinical conditions that affect a wide range of animal species, and primarily ruminants. *T. gondii* is considered one of the main abortigenic pathogens of small ruminants and can also cause extremely serious diseases in other domestic (e.g. cats and dogs) and wild (e.g. marine mammals, hares) species, leading to severe forms of encephalitis with a high fatality rate (Dubey et al. 2004).

*Neospora caninum* is also the most important abortigenic protozoa on dairy cattle farms worldwide. It is increasingly impacting small ruminants (sheep and goats), potentially causing a significant miscarriage rate (Rodrigues et al. 2020; Romanelli et al. 2021) and in alpaca (Serrano-Martínez et al. 2007). *T. gondii* also plays a significant role in public health as it is a zoonotic agent of major concern (Dini et al. 2023).

In addition to the clinical disorders associated with these infections, dogs contribute to the transmission and dissemination of both parasites in nature, becoming part of their epidemiological circuit in various ways, such as acting as predators for *T. gondii* or as vectors for the resistant forms of *N. caninum*.

This review compares *T. gondii* and *N. caninum* infections in dogs. These two diseases are often considered to be closely related in clinical settings. However, they exhibit numerous distinct epidemiological, clinical, diagnostic, and therapeutic response characteristics which should be clearly differentiated to support timely diagnosis and to establish effective therapeutic protocols and control strategies.

## Morphobiological aspects and routes of transmission

*Toxoplasma gondii* and *N. caninum* are two facultative heteroxenous parasites with particular biological cycles and several routes of transmission. Both parasites exhibit definitive and intermediate hosts at the same time (defined as complete host), harbouring both the sexual and asexual phases of the cycle, and they have also a wide range of alternative intermediate hosts.

Domestic cats (*Felis catus*) and numerous wild cats are recognised as definitive/complete hosts of *T. gondii*, and numerous domestic and wild animals are intermediate hosts (more than 200 species among mammals and birds), including dogs and humans (Hill et al. 2005; Al-Malki 2021). *Neospora caninum* has a more limited spectrum of intermediate hosts, consisting of wild ruminants (e.g. cervids) and domestic ruminants, camelids, equids and rodents. The domestic dog (*Canis familiaris*) and some wild canids, such as dingo (*Canis lupus*) and coyote (*Canis latrans*), are definitive and complete hosts (Cedillo et al. 2008).

*Neospora caninum* and *T. gondii* show the same biological and infection stages (i.e. oocysts, bradyzoites and tachyzoites), as well as very similar routes of transmission. However, they also have some distinctive features which are summarized in Table 1.

**Table 1:** Transmission routes differences between *Neospora caninum* and *Toxoplasma gondii* in dogs

	Toxoplasma gondii	Neospora caninum
Pre-patency time	N. A	Between 5 and 13 days post infection in experimental conditions
Patency time	N. A	Up to 27 days in experimental conditions
Excretion intensity	N. A	Not defined
Predominant route of infection	Horizontal	Vertical
Post-infection reactivation	Rare	Frequent
Transmission circuit	Domestic and wild	Domestic

*N. A* Not Applicable

Oocysts, defined as *Cystoisospora*-like due to their internal arrangement in two sporocysts each containing four sporozoites, are the small elements that the definitive host spreads in the environment through faeces and are responsible for transmission via the faecal-oral route. The oocysts shed are not directly infectious, and sporulate rapidly in the external environment within 24–72 h. Tachyzoites are the rapidly replicating morphotypes that spread during the acute phase of infection through the body of the intermediate hosts, including dogs. They are able to colonize any metabolically active cell (e.g. neurons, macrophages, hepatocytes, fibroblasts, muscle fibre cells, vessel endothelium cells, renal tubular epithelium cells, etc.) and show elective affinity for highly vascularized organs where parasite clusters at the pseudocyst stage.

**Fig. 1:**
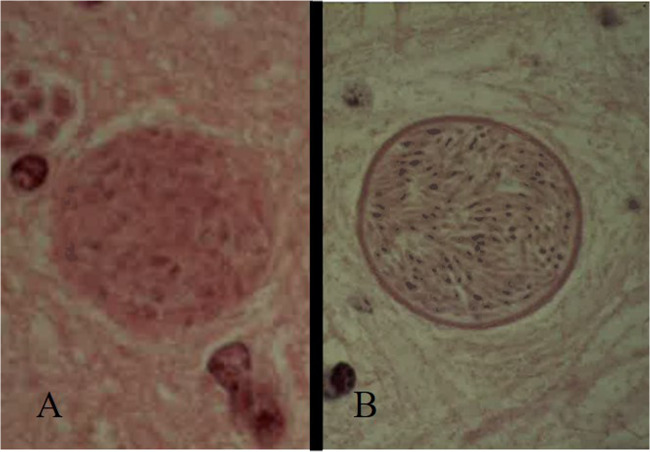
Tissue cysts of *Toxoplasma gondii* (**A**) and *Neospora caninum* (**B**). The wall is thicker in *N. caninum* (1–4 μm), then in *T. gondii* (< 0.5 μm)

Bradyzoites replicate slowly and are typical of the chronic phase of infection, which develop within tissue cysts reaching the size of 70–100 μm at full development (within three months after infection). The thickness of the wall differs between the two parasites, which is thicker in *N. caninum* (1–4 μm), then in *T. gondii* (< 0.5 μm) (Dubey et al. 2002) (Fig. 1). The cysts develop in anatomical sites that are hard to access immunologically. In the case of *N. caninum*, they develop exclusively in the neural tissues, whereas in the case of *T. gondii* cysts can be found in many organs, including voluntary and involuntary striated muscles. Under conditions of immunosuppression or certain physiological situations (e.g. pregnancy), the cysts may rupture, releasing the bradyzoites, which replicate rapidly as tachyzoites and spread through the blood to the placenta and *foetus* (in same species) and in other organs (Silva and Machado 2016; Sanchez and Besteiro 2021).

The reactivation skills of the chronic phases of the infection are different for *T. gondii* and *N. caninum*. After the first infection, in fact *T. gondii* can evoke a fully protective interferon-γ mediated immunity, which may be broken only in rare cases following extremely serious and immunosuppressive underlying diseases (e.g. canine distemper virus or leishmaniosis) (Moretti et al. 2006). On the other hand, *N. caninum* can be reactivated easily, even after hormonal disorders that are physiologically related to the oestrous cycle (Silva and Machado 2016).

Concerning the routes of transmission, both *N. caninum* and *T. gondii* can be transmitted from the definitive host to an intermediate host and vice-versa through a faecal-oral and prey-predator circuit, respectively. They can also be transmitted from an intermediate host to another intermediate host through carnivorism and from a definitive host to another definitive host by contaminated food or water containing oocysts (Silva and Machado 2016; Sanchez and Besteiro 2021).

In dogs, *T. gondii* and *N. caninum* infection are acquired via three routes of transmission: (i) ingestion of sporulated oocysts present in the soil and which contaminate food and water; (ii) ingestion of tissue cysts containing bradyzoites through the consumption of raw or undercooked meat or as a result of predation; (iii) transmission of tachyzoites through the placenta (Dubey 2008; King et al. 2011). These routes of transmission have different frequencies in dogs: *T. gondii* is acquired mainly horizontally, through predation, while *N. caninum* is mainly transmitted vertically, through the maternal-foetal lineage (Barber and Trees 1998; Machacova et al. 2016).

### Epidemiology and risk factors

*Toxoplasma gondii* and *N. caninum* infections are found globally in dog populations, with variable prevalence rates according to the sample population, the risk factors considered and the accuracy of the diagnostic tests used (Dubey et al. 2020). Recent data available on *T. gondii* and *N. caninum* seroprevalence in dogs in various parts of the world may thus diverge (Tables 2 and 3).

**Table 2:** Trends in *Toxoplasma gondii* prevalence in dogs over the past 20 years

Country	Type	No. tested	*N*. positive	% positive	Test	Cut off	References
Angola	Pets, clinic	103	16	15.5	MAT	1/20	Lopes et al. 2014
Egypt	Strays	51	50	98.0	MAT	1/5	El Behairy et al. 2013
Nigeria	Slaughterhouses	278	55	19.8	MAT	n.a	Ayinmode et al. 2016
Senegal	Pets, strays	100	68	68.0	MAT	1/40	Kamga-Waladjo et al. 2013
Gabon	Strays	70	43	61.4	MAT	1/20	Mercier et al. 2010
Brazil	Strays	325	169	52.0	IFAT	1/16	Minervino et al. 2012
Brazil	Pets, clinic	384	37	9.6	IFAT	1/16	Brasil et al. 2018
Brazil	Kennels	364	175	48	IFAT	1/16	Brasil et al. 2018
Brazil	Clinic	598	259	43.4	IFAT	1/16	Pinto-Ferreira et al. 2016
Brazil	Pets	729	119	16.3	IFAT	1/16	Benitez et al. 2017
Brazil	City and rural	649	265	40.8	IFAT	1/16	Pinto-Ferreira et al. 2019
Brazil	Pets	320	156	48.75	IFAT	1/16	Magalhães et al. 2017
Brazil	Pets	264	21	7.95	IFAT	1/16	Da Cunha et al. 2020
Cuba		176	128	72.7	ELISA	n.a	Navarrete et al. 2017
Grenada, Caribbean	Strays	249	89	35.7	MAT	1/25	Dubey et al. 2013
USA	Pets	14	6	42.8	MAT	1/25	Verma et al. 2016
USA	Kennels	90	19	21.0	IFAT	1/50	Rosypal et al. 2010
Mexico	Rural, stray and pets	210	123	59	IFAT	1/16	Cruz-Vázquez et al. 2023
Mexico	Stray dogs	154	95	61.7	ELISA	n.a	Cedillo-Peláez et al. 2012
Mexico	Animal shelter	101	68	67.3	MAT	1/25	Alvarado-Esquivel et al. 2014
China	Pets	1876	467	24.9	ELISA	n.a	Cui et al. 2012
China	Slaughterhouses	364	189	51.9	ELISA	n.a	Jiang et al. 2015
China	Pets	1736	56	3.2	IHA	1/64	Wang et al. 2011
China	Pets	408	37	9.1	ELISA	n.a	Jiang et al. 2016
China	Pets	314	11	3.5	IHA	1/64	Li et al. 2012
China	Pets	611	132	21.6	IHA	1/64	Duan et al. 2012
China	Stray or free-living	231	93	40.3	ELISA	n.a	Yan et al. 2012
China	Pet hospitals and slaughterhouses	341	15	4.4	MAT	1/25	Zhu et al. 2022
Japan	Kennel	325	6	1.9	LAT	1/16	Oi et al. 2015
Pakistan	Pets	408	116	28.4	ELISA	n.a	Ahmad et al. 2014
Thailand	Strays	230	25	10.9	LAT	1/64	Jittapalapong et al. 2009
Iran	Pets	180	84	46.6	ELISA	n.a	Zarra-Nezhad et al. 2017
Turkey	Strays	107	58	54.0	DT	1/16	Şahal et al. 2009
Turkey	Pets	179	172	96.1	DT	n.a	Gicik et al. 2010
Italia	Hunting dogs	398	94	24	IFAT	1/50	Machacova et al. 2016
Italia	Pets	114	64	56.1	IFAT	1/20	Macrì et al. 2009
Italia	Pets, hunting dogs, truffle dogs and watchdogs	120	35	29.2	IFAT	1/40	Dini et al. 2024
Poland	Pets	17	9	63	MAT	n.a	Sroka and Szymańska 2012
Portugal	Pets	673	256	38	MAT	1/20	Lopes et al. 2011
Spain	Pets, clinic	769	235	30.6	MAT	1/25	Cano-Terriza et al. 2016
UK	Ocular and not-ocular disorders	135	9	6.7	LAT	1/64	Kosec et al. 2019
UK	Dogs with neurological signs	201	8	3.98	IFAT	1/50	Coelho et al. 2019

*ELISA* Enzyme-linked immunosorbent assay, *IH A* Indirect hemagglutination antibody test, *IFAT* Indirect fluorescent antibody test, *LAT* Latex agglutination test, *MAT* Modified agglutination test, *n. a* not available

**Table 3:** Trends in *Neospora caninum* prevalence in dogs over the past 20 years

Country	Type	No. tested	*N*. positive	% positive	Test	Cut off	References
Brazil	Rural	37	25	67.6	IFAT	1/50	Oliveira et al. 2004
Brazil	Urban	156	4	2.6	IFAT	1/50	Sicupira et al. 2012
Brazil	Pets	154	3	1.9	IFAT	1/50	Campos et al. 2022
China	Slaughter houses	96	6	6.25	MAT	1/25	Yang et al. 2014
China	Domestic dogs	1176	172	15	IFAT	1/50	Wang et al. 2016
China	Domestic dogs	476	95	20	IFAT	1/50	Gao and Wang 2019
China	Stray dogs	224	145	64.7	ELISA	n.a	Yang et al. 2022
Pakistan	Farm, pet and stray dogs	600	141	23.5	ELISA	n.a	Nazir et al. 2014
Indonesia		147	5	3.4	ELISA	n.a	Dwinata et al. 2018
Iran	Flock and stray dogs	288	14	4.86	IFAT	1/50	Adhami et al. 2020
Australia	Clinics	192	27	14.1	IFAT	1/50	Barker et al. 2021
Turkey	House and stray dogs	187	31	16.6	ELISA	n.a	Zhou et al. 2016
Italy	Farm dogs and breeding facilities	100	32	32	IFAT	1/50	Robbe et al. 2016
Italy	Pets	707	77	10.9	ELISA	n.a	Capelli et al. 2004
Germany	Breeding bitches	218	16	7.33	ELISA	n.a	Villagra-Blanco et al. 2018
Poland	Clinics	257	56	21.7	ELISA and WB	n.a	Goździk et al. 2011
Albania	Pets	602	110	18.3	IFA	1/50	Hamel et al. 2016
Portugal	Domestic and stray dogs	441	35	7.9	ELISA	n.a	Maia et al. 2014
Portugal		286	93	32.5	IFA	1/50	Waap et al. 2017
Serbia		99	17	17.2	IFAT	1/50	Kuruca et al. 2013
UK	Dogs with neurological signs	201	14	6.96	IFAT	1/50	Coelho et al. 2019

*ELISA* Enzyme-linked immunosorbent assay, *IFAT* Indirect fluorescent antibody test, *MAT* Modified agglutination test, *WB* Western Blot

*n. a*  not available

The seroprevalence of *T. gondii* increases with the age of the animals, due to exposure over time to potential horizontal routes of infection (e.g. faecal-oral contamination and predatory activity). The habitat and type of diet administered are considered as risk factors. Working dogs (e.g. hunting dogs) in wild environments or those living in rural or semirural areas are exposed to a greater extent, since they are most likely to carry out predatory activities (Lopes et al. 2011; Cano-Terriza et al. 2016). In addition, animals fed with raw meat or household food are considered at risk (Ali et al. 2003; Lopes et al. 2011). Cohabiting with cats that live in semi-freedom may be also considered a risk factor, above all for dogs exhibiting coprophagia behaviour (Dini et al. 2024).

Regarding the morbidity of *T. gondii* infection, an extremely important factor is the presence of viral or bacterial coinfections (e.g. canine distemper virus, *Ehrlichia canis*) (Headley et al. 2013; Cardinot et al. 2016) that have an immunosuppressive effect. In fact, dogs rarely manifest clinical signs of primary toxoplasmosis, but more frequently show secondary reactivation forms in adult animals (Webb et al. 2005).

*N. caninum* seropositivity rates do not increase significantly with the age of the animals, supporting the hypothesis that the ingestion of sporulated oocysts is not so important in the epidemiological circuits as their elimination is extremely rare and mostly only demonstrated experimentally (Dubey and Schares 2011). Young animals (i.e. puppies) are more susceptible to *N. caninum*, since vertical transmission is more prevalent than in *T. gondii*. Infection is more common in some breeds (e.g. German Shepherd, Alsatian, Labrador, Golden Retriever, Basset Hound and Greyhound), which is not related to a real genetic predisposition, but to the establishment of infected maternal-filial lines on breeding farms (Dubey et al. 2009).

Dogs on dairy farms are considered to be at high risk, because they may ingest placental and aborted foetuses containing the parasite, as well as animals with an outdoor lifestyle prone to predatory activities. In general, all animals that could potentially ingest tissues of intermediate hosts containing pseudocysts and cysts are considered at neosporosis risk (Paradies et al. 2007; Dubey et al. 2009).

The epidemiological role that dogs play in the transmission of *T. gondii* is more marginal than that of cats because they do not act as a definitive host, and they are unlikely to be preyed upon. However, dogs can act as mechanical carriers of *T. gondii* oocysts, excreting them in their faeces after ingestion from cat stools (Schares et al. 2005). Moreover, *T. gondii* oocysts can contaminate dog fur, potentially leading to human infection through contact with the dog’s coat, mouth, and feet (Lindsay et al. 1997; Dubey et al. 2020). In contrast, through the elimination of oocysts of *N. caninum*, the dog is considered as the main animal responsible for the outbreak of high-prevalence abortions (i.e. abortion storms) on dairy farms (Bartels et al. 1999).

Regarding the potential zoonotic transmission of *T. gondii*, symptomatic dogs, especially those with respiratory forms, may act as a source of infection for humans. This can occur through the *sputum* containing tachyzoites, which can penetrate damaged skin or mucous membranes of people handling them (e.g. veterinary surgeons performing medical or necropsy procedures) or those in close contact with them (Tenter et al. 2000). In contrast, dogs do not seem to show a zoonotic risk for humans concerning *N. caninum*, as no zoonotic value has been confirmed to date. However, some reports have shown varying degrees of human exposure to *N. caninum* through antibody assays (Tranas et al. 1999; Duarte et al. 2020). Additionally, experimental studies have demonstrated possible vertical transmission in non-human primates, resulting in fatal encephalitis that resembles that induced by *T. gondii* (Barr et al. 1994).

## Pathogenesis and clinical expression

*Toxoplasma gondii* and *N. caninum* infections in dogs evolve mainly in an asymptomatic form. However, when clinical manifestations occur, morbidity and mortality rates depend on several factors, primarily the animal’s age and immune *status.* Additionally, the transmission route (vertical *versus* horizontal) and the stage of infection (acute, chronic, or reactivation) are significant determinants (Carruthers and Suzuki 2007; Dubey et al. 2009; Swinger et al. 2009). As observed in other species, including humans and rodents, the severity of the disease and the various clinical presentations in dogs may also correlate with the infectious dose. No evidence currently exists on a possible correlation between parasite lineage and clinical forms in either *T. gondii* or *N. caninum* (Calero-Bernal and Gennari 2019).

Acquired acute forms related to post-natal primary infection (more common in *T. gondii* than in *N. caninum*), reactivation chronic forms and congenital forms have been described (Dubey and Lindsay 1996; Montoya and Liesenfeld 2004).

During the acute phase of infection, *T. gondii* and *N. caninum* exhibit pathogenic effects through active invasion and replication at the multiorgan intracellular level by tachyzoites. This phase is characterized by cell lysis and subsequent inflammatory and necrotic processes. In contrast, the chronic phase is characterized by general inactivity of both the immune system and organ responses to the parasites. Bradyzoites are located in tissue cysts within the central and peripheral nervous system for *N. caninum*, and in the CNS and muscles for *T. gondii*, without causing damage. Reactivation occurs when there is an immune breakdown due to immunosuppressive conditions, which can lead to renewed local replication of tachyzoites, potentially resulting in severe organ damage or, more rarely, systemic dissemination (Silva and Machado 2016; Sanchez and Besteiro 2021).

Additionally, congenital clinical disorders can occur when tachyzoites cross the placental barrier and replicate at the multiorgan level in the foetus (Silva and Machado 2016; Sanchez and Besteiro 2021).

The clinical conditions most associated with toxoplasmosis and neosporosis in dogs include peripheral and central neurological disorders, reproductive illnesses, dermatological problems and systemic disorders (Ruehlmann et al. 1995; Dubey and Lindsay 1996; Dubey et al. 2009; Calero-Bernal and Gennari 2019; Barker et al. 2021). Behavioural changes have also been reported in dogs and wild canids affected by toxoplasmosis (Papini et al. 2009; Milne et al. 2020).

## Peripheral neurological disorders

**Fig. 2:**
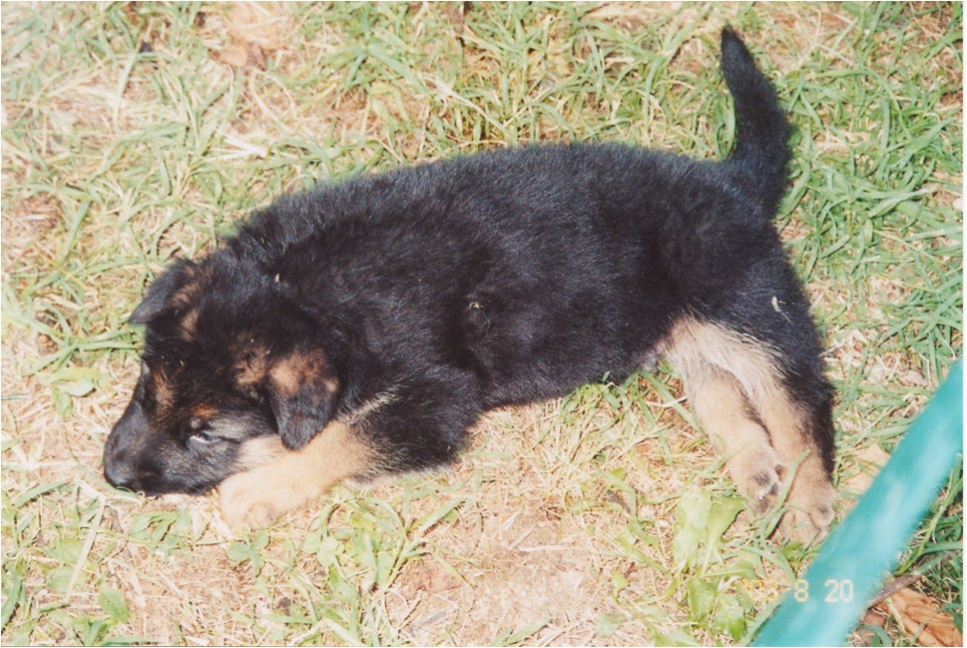
An 8-week-old German Shepherd puppy affected by *Neospora caninum* exhibits characteristic stiff hyperextension (arthrogryposis) of both hind legs

Peripheral neurological disorders are more frequently related to *N. caninum* infections than to *T. gondii* and include polyradiculoneuritis associated with chronic subacute evolving myositis. They are observed mainly in puppies under six months of age that have acquired the infection via the congenital route (Lyon 2010). Clinical signs may be evident at birth, but are observed more frequently in the first 5–8 weeks of life (Lyon 2010). In the starting phase, the parasite affects the lumbosacral plexus, consequently neurological signs appear in the hind legs. Ipsilateral muscle weakness is observed, which rapidly affects the contralateral leg, and is followed by a rapid neurogenic atrophy with paraplegia and typical stiff hyperextension (arthrogryposis) of one or both hind legs (Dubey et al. 2009) (Fig. 2). In this phase, the animal retains deep pain perception and consciousness. This condition may progress to a chronic state without further developments. However, medium to large-sized animals are often euthanized due to poor quality of life and the difficulties in their management by owners.

In some cases, *N. caninum* can even affect the nerve roots of the forelimbs and CNS, causing tetraplegia and impairments of the brainstem and cranial nerves. This results in sensory deficits, nystagmus, absence of a menace response, dysphagia, megaesophagus, and arrhythmias, which may lead to sudden death due to cardiac arrest or phrenic nerve blockage accompanied by respiratory difficulties (Basso et al. 2005; Dubey et al. 2009). Additionally, reduced tail movement, perineal sensitivity deficits, and local faecal and/or urinary incontinence have been reported (Basso et al. 2005). The involvement of CNS is generally considered a negative prognostic indicator.

To date, only one case of suspected toxoplasmosis in a 12-week-old puppy has been reported in the literature, presenting with hindlimb weakness, sarcopenia, rapidly progressing ascending paralysis, and respiratory distress (Chen et al. 2023).

## Central neurological disorders

Central neurological disorders caused by *T. gondii* and *N. caninum* are more frequently observed in animals older than one year. The neurological signs include sequelae of chronic infections. Specifically, the reactivation of latent cysts in the brain can cause focal or multifocal inflammatory processes depending on the selective tropism that the two parasites exhibit for different parts of the CNS (Carruthers and Suzuki 2007; Silva and Machado 2016). *Toxoplasma gondii* may cause behavioural alterations, circling, tremors, and cranial nerve deficits. *N. caninum* infections are more often associated with reduced levels of consciousness, head shaking, cerebellar ataxia, hypermetria, and cervical hyperesthesia, which are manifestations of meningoencephalitis and necrotizing cerebellitis (Dubey and Lindsay 1996; Garosi et al. 2010; Calero-Bernal and Gennari 2019; Didiano et al. 2020).

*Toxoplasma gondii* and, less frequently, *N. caninum*, are included in differential diagnoses of epilepsy, probably due to evidence in studies on mouse models and on several case-control studies conducted on humans (Ferguson and Hutchison 1987; Sadeghi et al. 2019). Recently, a study searched the medical record database of the Veterinary Teaching Hospital, University of Perugia (Italy) for dogs serologically tested by IFAT for *T. gondii* and *N. caninum* between 2017 and 2023. In order to investigate the serological correlation between these pathogens and epilepsy, the dogs were stratified by a clinical diagnosis of epilepsy or suffering from different conditions. The results obtained do not seem to support the role of *T. gondii* and *N. caninum* as causal agents of epilepsy in dogs (Morganti et al. 2024).

## Reproductive disorders

*Toxoplasma gondii* and *Neospora caninum* are known to cause abortions or stillbirths. However, there is no clear evidence supporting the role of these protozoa in reproductive disorders in dogs, which is largely inferred from their pathogenicity in other animal species (e.g., sheep and cattle). While *N. caninum* is primarily transmitted vertically in dogs, this transmission rarely results in foetal death (Reichel et al. 2007). Unlike *T. gondii*,* N. caninum* can reactivate and cross the placental barrier during gestation, as it does not elicit a strong cell-mediated immune response (Barber and Trees 1998). This transmission can lead to the onset of polyradiculoneuritis in varying numbers of puppies within a few weeks after birth. Asymptomatic puppies, particularly females used for breeding, can perpetuate infected bloodlines within breeding farms. The efficiency of *T. gondii* transplacental transmission in dogs is lower than that of *N. caninum*. However, there is a stronger correlation between *T. gondii* infection and instances of abortion and stillbirth (Bresciani et al. 2009). Puppies born with congenital toxoplasmosis often present without vital signs and exhibit multiple organ syndromes, with neurological manifestations such as encephalitis accompanied by hepatomegaly, ascites, interstitial pneumonia, and myocarditis (Calero-Bernal and Gennari 2019).

### Dermatological forms

Cutaneous clinical signs are associated mainly with *N. caninum.* Adult dogs with neosporosis in the case of concomitant diseases or long corticosteroid treatment may present with dermatological lesions characterized by erythematous epidermal nodules of various sizes (from 0.5 to 5 cm) and a tendency for ulcers. This clinical picture may be accompanied by satellite lymphadenomegaly. The histopathological lesions are described as pyogranulomatous and necrotizing dermatitis and alopecia, panniculitis with multifocal vasculitis, and vascular thrombosis (Webb et al. 2005; Park et al. 2007; Amir et al. 2008; Hoffmann et al. 2012).

Cases of systemic toxoplasmosis in dogs with diffusion of tachyzoites in the skin have been found after immunosuppressive treatments with corticosteroids or transplants (Bernsteen et al. 1999; Hoffmann et al. 2012). However, dermatological forms caused by *T. gondii* are reported above all in humans and rarely in immunocompromised cats (Mawhorter et al. 1992; Beatty and Barrs 2003).

## Other clinical forms

Other clinical forms associated with toxoplasmosis and neosporosis in dogs consist of myocarditis, hepatitis, pancreatitis and interstitial pneumonia, the latter being associated above all with *T. gondii* infections (Calero-Bernal and Gennari 2019; Dorsch et al. 2022). Although less frequently than observed in cats, *T. gondii* may also cause eye diseases such as necrotizing conjunctivitis (Swinger et al. 2009), anterior uveitis, endophthalmitis and chorioretinitis in dogs (Wolfer and Grahn 1996).

## Behavioural changes

Although the literature reports that *T. gondii* is implicated in behavioural modification in rodents and humans (Johnson and Koshy 2020; Tyebji et al. 2019), similar evidence is lacking for other hosts, including dogs. A single case report found a sudden noise sensitivity described as behavioural change in an 8-year-old female collie infected with *T. gondii* (Papini et al. 2009).

More evidence of correlations between *T. gondii* and behavioural changes has been found for wild canids. Recent studies report many wild red foxes exhibiting a range of aberrant behavioural traits, including apparent lack of fear and increased affection, and suggested that the infection with *T. gondii* and likely co-infection with Fox *Circovirus* and/or another neurotropic agent could be implicated in this spectrum, subsequently classified as Dopey Fox Syndrome (DFS) (Milne et al. 2020). A study conducted in Yellowstone National Park (USA) found that seropositive wolves were more likely to make high-risk decisions such as dispersing and becoming a pack leader, both of which factors are critical to individual fitness and wolf vital rates.

These findings demonstrate that parasites have important implications for intermediate hosts, beyond acute infections, through behavioural impacts (Meyer et al. 2022). Similarly, Gering et al. (2021) found that, after three decades of field observations, *T. gondii*-infected hyena cubs approach lions more closely than uninfected peers and thus have higher rates of mortality. No data on behavioural changes and infection by *N. caninum* have been reported in dogs or wild canids.

### Diagnosis

Toxoplasmosis and neosporosis can be clinically confused with each other and with other diseases. A precise clinical examination supported by numerous specific (i.e., parasitological) and non-specific tests is therefore essential. Table 4 highlights the primary specific and non-specific tests that aid in diagnosing the various clinical manifestations of toxoplasmosis and neosporosis in dogs.

**Table 4:**
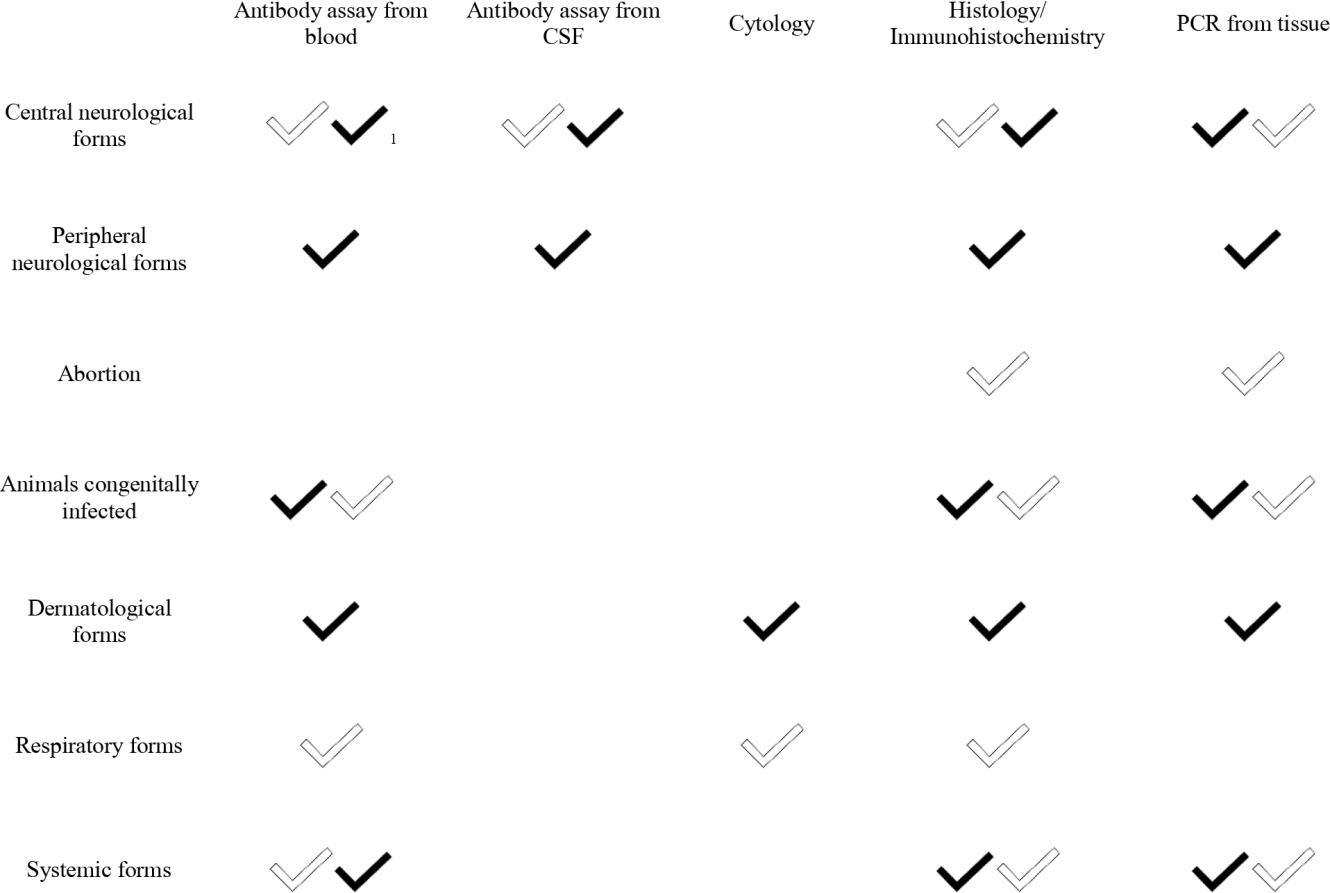
Specific and nonspecific exams to support the diagnosis of the different clinical forms of canine toxoplasmosis and neosporosis

^1^*Toxoplasma gondii* = white tick; *Neospora caninum* = black tick

### Specific tests

The diagnosis of *T. gondii* or *N. caninum* infections requires a series of tests that encompass both direct methods (which detect the parasite within lesions), and indirect methods that detect the host’s immune response. Indirect methods are predominantly used, especially in the initial stages, and typically involve screening tests.

**Fig. 3:**
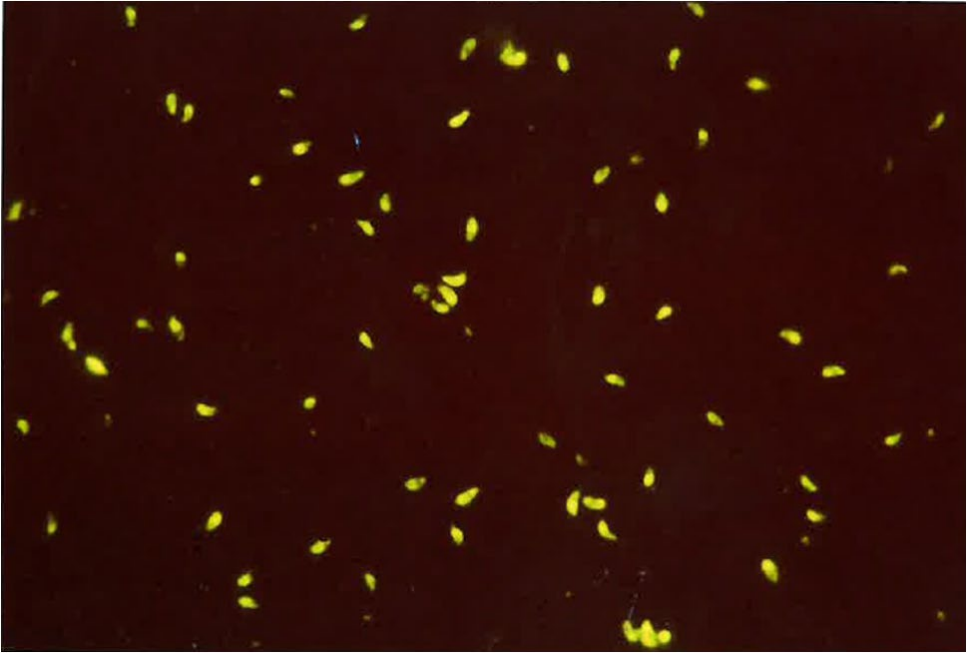
Indirect fluorescent antibody test used for the diagnosis of *Toxoplasma gondii* (cut off 1/40)

The most commonly employed immunodiagnostic tests in dogs include the Indirect Fluorescent Antibody Test (IFAT), which is known for its sensitivity and specificity (Fig. 3). For *N. caninum*, the IFAT typically utilizes a cut off of 1/50, which for *T. gondii*, is 1/40 (Silva et al. 2007; Dini et al. 2023). The Enzyme Linked Immuno Assay (ELISA) is also used. Both tests detect IgG and IgM antibodies generated against either corpuscular antigens (IFAT) or soluble antigens (ELISA) of varying degrees of purification.

The serological result must be interpreted with caution, since both infections are persistent and have a low degree of morbidity. Seropositivity may therefore demonstrate infection and not the active role of the parasite in the disease progression. Low antibody titres (i.e. IFAT results lower than the cut off dilution) or apical reactions (i.e. reactions limited to the apex of the parasite) may be regarded as the expression of cross-reactivity with parasites of the Sarcocystidae family (i.e. *Sarcocystis*, *Hammondia*, which are closely antigenically correlated (Gondim et al. 2017), or with other common pathogens affecting dogs such as *Leishmania infantum* and *Ehrlichia canis* (Silva et al. 2007; Zanette et al. 2014).

Both IgM and IgG antibodies need to be assessed because IgM antibodies can be detected starting from the second week after the infection and persist for about four months, while IgG antibodies are produced starting from the third to fourth week after the infection and may persist throughout out life at the cut off level (Sinnott et al. 2017). In addition, in congenital forms, caused especially by *N. caninum*, the interference of maternal passive immunity in the first 14 weeks of life can complicate the diagnosis. In this case, the detection of IgM is mandatory (Anderson et al. 2000).

In symptomatic animals, it is useful to distinguish the classes of antibodies in order to assess the possibility of a recent infection and to define the antibody titre. In fact, active infections are generally associated with high antibody titres (IgG starting from 1/200 for *N. caninum* and 1/80 for *T. gondii*). Nevertheless, since a low antibody titre does not rule out active infection, seroconversion should be verified after two weeks in the case of a well-founded suspicion along with histological or immunohistochemical tests (Piergili Fioretti 2004; Sinnott et al. 2017).

Serological positivity has a reduced positive predictive value in relation to both central and peripheral neurological forms. In these disorders, the antibody titre in the cerebrospinal fluid (CSF) is considered more accurate. However, the possibility of false positives related to contamination of the blood during collection remains, as well as the normal passive transfer of antibodies through the blood-brain barrier. Demonstration of intrathecal antibody production entails establishing the ratio between the titres of antibodies in the CSF and circulating antibodies (QIgG = Cerebrospinal fluid IgG/Serum IgG) (Whitney Marlyn and Ripley Coates 2020).

Direct methods often have limitations related to *intra-vitam* sampling. These tests include (a) cytological tests for tachyzoite identification prepared from dermatological lesions or in bronchoalveolar washing and CSF in the course of interstitial pneumonia and neurological disorders, respectively; (b) histological examinations on biopsy samples of CNS (e.g. tibial nerve during neosporosis), muscles, liver, spleen, heart, lungs, skeletal muscles and kidneys to reveal cystic and pseudocyst formations. These are the only examinations that establish a cause-effect connection with the lesions, however, they are difficult to apply *intra-vitam* due to the invasiveness of the sampling.

Notably, the cysts of *N. caninum* and *T. gondii* have the same size (100 μm), and only differ in the thickness of the wall (thicker in *N. caninum* than in *T. gondii*) and may have a range of variability that depends on the phase of development. Diagnostic certainty therefore requires PCR tests using various protocols (e.g. traditional PCR, nested-PCR, real-time PCR) or by immunohistochemical target (IHC), which detects immunodominant surface antigens. Although PCR offers a more accurate means of revealing active infection by amplifying parasite-specific deoxyribonucleic acid in CSF, false negatives are possible if the protozoan is embedded deeply into the tissues (Coelho et al. 2019).

Direct tests also include coprological examinations to identify the presence of *N. caninum* oocysts. However, their detection in dogs is rare and does not necessarily correlate with clinical manifestations associated with the parasite’s extraintestinal cycle. From an epidemiological standpoint, detection of oocysts in faeces can help to determine the role of dogs as a potential source of infection in dairy farms. The oocysts can be detected using a flotation technique with a 33% zinc sulphate solution. Despite their infrequent detection, oocysts of *N. caninum* resemble those of *Cystoisospora*, making them potentially confusable with those from other dog coccidia or incidental parasites acquired through predation on birds and rodents. Table 5 presents the main morphological characteristics that differentiate *N. caninum* oocysts from those of other species.

**Table 5:** Morphological characteristics of oocysts belonging to Apicomplexa Phylum

		Toxoplasma gondii	Neospora caninum	Cystoisospora spp.	Sarcocystis spp.	Hammondia spp.	Cryptosporidium spp.
Oocysts sizes	Medium (25–35 μm) and large (35–45 μm) size			x			
	Small size (10–15 μm)	x	x		x	x	
	Very small size (5–10 μm)						x
Stage of development at the shedding time	Sporulated				x		x
	Not sporulated	x	x	x		x	
Micropyle	Presence						
	Absence	x	x	x	x	x	x

### Non-specific tests

Non-specific tests may support diagnosis and identify the degree of impairment of different organs. These include (a) complete blood count and biochemical blood tests, useful above all in systemic disorders and polyradiculoneuritis; (b) total protein concentration and cytological analysis of the CSF in central and peripheral neurological forms; (c) X-ray examination of chest and magnetic resonance imaging for interstitial pneumonia and central neurological disorders, respectively. Table 6 indicates the most frequently detectable alterations recorded in dogs affected by toxoplasmosis and/or neosporosis.

**Table 6:** Detectable alterations in nonspecific tests during neosporosis and toxoplasmosis in dogs

Non specific test	Neosporosis	Toxoplasmosis	References
Complete blood count and leukocyte formula	Variable patterns: from no alterations to mild normochromic normocytic anemia associated with moderate neutrophilic leukocytosis	Non regenerative anemia Leukocytosis	(Dubey et al. 2009; Migliore et al. 2017; Borges-Silva et al. 2021)
Biochemical analysis	Increased CK, LDH, ALT and AST Decrease in total proteins	Increased total bilirubin, ALT, and AST Hypoproteinemia and hypoalbuminemia	(Parzefall et al. 2014; Curtis et al. 2020; Didiano et al. 2020)
Biochemical and cytometric examination of CSF	Mildly to markedly increased total nuclear cell count (sometimes > 1000 cells/µl) Predominantly/occasionally mixed mononuclear pleocytosis (neutrophils or eosinophils) Mildly to markedly increased protein concentration (positive Pandy reaction)	Pleocytosis with a mixed cell population of neutrophils and macrophages Tachyzoites may be observed	(Dubey et al. 2009; Didiano et al. 2020; Borges-Silva et al. 2021)
Chest X-ray	Interstitial patterns	Interstitial patterns	(Greig et al. 1995; Pepper et al. 2019)
Brain MRI	Cerebellar atrophy Multifocal granulomatous lesions in the brain (multiple areas of interest)	No data reported	(Parzefall et al. 2014; Didiano et al. 2020)

*CSF* Cerebrospinal fluid, *MRI* Magnetic Resonance Imaging

### Treatment and prevention

No treatment is required in asymptomatic dogs affected by *T. gondii* and *N. caninum* infections. In addition, no currently available molecule is able to penetrate the wall of the cyst and thus eliminate the bradyzoites in chronic forms. In contrast, dogs with clinical signs require prompt treatment in order to suppress the replication of tachyzoites and prevent clinical progression.

Concerning toxoplasmosis, clindamycin is the elective drug at a dosage of 12.5–25 mg/kg orally, twice a day, for at least 4 weeks, however, in some cases, 2 months of treatment may be required. Clindamycin administered *per os* may cause anorexia, vomiting and diarrhoea in dogs treated at high doses, as it irritates the gastrointestinal mucosa. Alternatively, a parenteral treatment can be administered, via the intramuscular route, with 25 mg/kg of clindamycin phosphate, twice a day, for 4 weeks or with a combination of trimethoprim-sulfadiazine at a dosage of 15–30 mg/kg, orally, twice a day, for 4–6 weeks.

In general, dogs with toxoplasmosis react better to treatment than those affected by clinical neosporosis. In fact, when muscle contracture has already set in during neosporosis, the administration of drugs is only partially effective. Neosporosis treatment consists in clindamycin at a dosage of 10 mg/kg, orally, three times a day, for 4 to 24 weeks or 7.5–25 mg/kg, orally, twice a day, for at least 4 weeks. Alternatively, a combination of trimethoprim and sulfadiazine at a dosage of 15–30 mg/kg, orally, twice a day, for at least 4 weeks, and pyrimethamine at a dosage of 1 mg/kg, orally, once a day, can be administered to exploit their synergy (Dubey and Lindsay 1996; Lyon 2010).

Regarding prophylactic measures, dogs should not be fed with cooked meat or entrails. Predatory activity should be prevented, together with access to cats’ litter boxes, especially for animals that practise coprophagia. Dogs should never have access to the placental materials of bovine or small ruminants, dead calves or lambs or foetal membranes. Effective vaccines to protect dogs and prevent infection are not commercially available for *N. caninum* or for *T. gondii*, however some attempts were made to design vaccine against *N. caninum* for use in dogs (Nishikawa et al. 2000).

Seropositive bitches may transmit *N. caninum* and, less frequently, *T. gondii* to their puppies. It is thus advisable to exclude them from any reproduction program, as they could generate infected maternal-filial lines, which could maintain the infection endemicity on breeding farms and in canine populations. Finally, immunosuppressive treatments in seropositive dogs should be avoided because they could be potentially responsible for reactivating the infection (Barker et al. 2021).

## Conclusions

Differentiating between a *T. gondii* and *N. caninum* infection in dogs is crucial for the individual animal’s health and also for preventing transmission to humans, and effectively managing diseases on farms. This differentiation also optimizes treatments and promotes enhanced public health and food safety measures.

## Data Availability

No datasets were generated or analysed during the current study.
